# Supplementary material for: Causal Relationship between Aging and Anorexia Nervosa: A White-Matter-Microstructure-Mediated Mendelian Randomization Analysis
Source: Biomedicines. 2024 Aug 16;12(8):1874. doi: 10.3390/biomedicines12081874 (PMC11351342; doi:10.3390/biomedicines12081874)
Supplement: Supplementary file 1 [file biomedicines-12-01874-s001.zip › Supplementary Figure/Figure S4/Singe SNP plot of MR analysis for TL to ubm-b-1661.pdf]

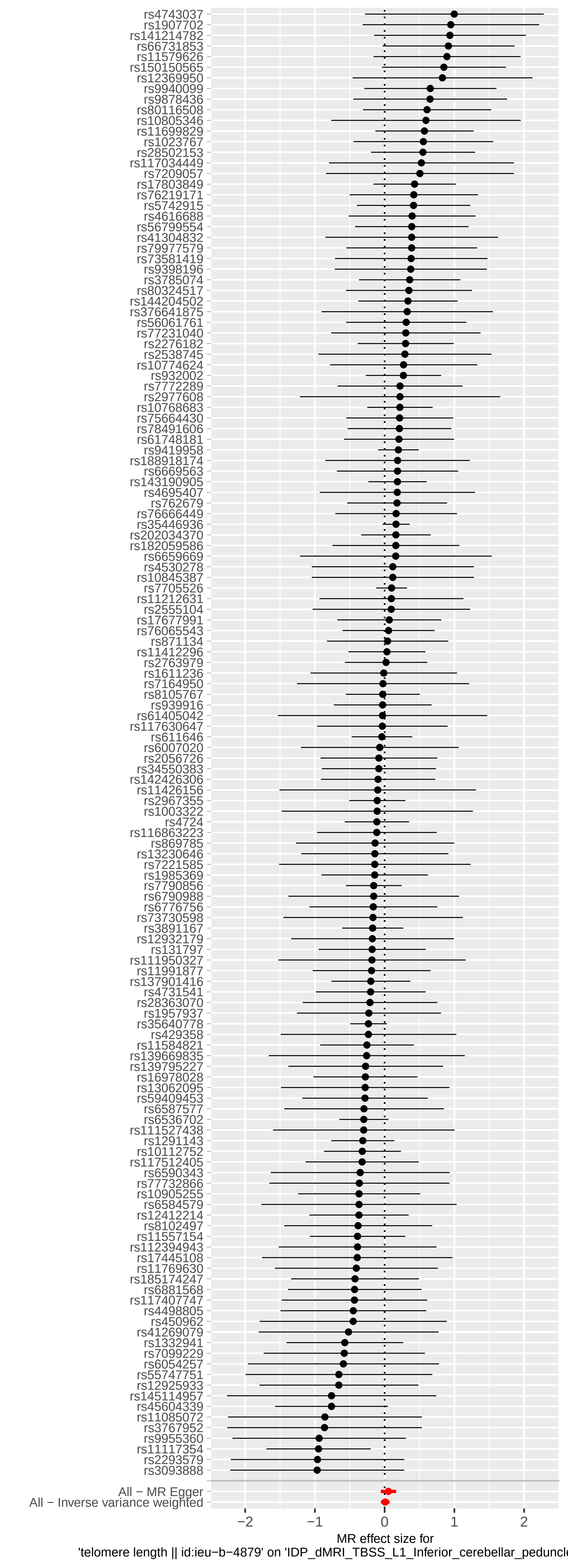
rs4743037
rs1907702
rs141214782
rs66731853
rs11579626
rs150150565
rs12369950
rs9940099
rs9878436
rs80116508
rs10805346
rs11699829
rs1023767
rs28502153
rs117034449
rs7209057
rs17803849
rs76219171
rs5742915
rs4616688
rs56799554
rs41304832
rs79977579
rs73581419
rs9398196
rs3785074
rs80324517
rs144204502
rs376641875
rs56061761
rs77231040
rs2276182
rs2538745
rs10774624
rs932002
rs7772289
rs2977608
rs10768683
rs75664430
rs78491606
rs61748181
rs9419958
rs188918174
rs6669563
rs143190905
rs4695407
rs762679
rs76666449
rs35446936
rs202034370
rs182059586
rs6659669
rs4530278
rs10845387
rs7705526
rs11212631
rs2555104
rs17677991
rs76065543
rs871134
rs11412296
rs2763979
rs1611236
rs7164950
rs8105767
rs939916
rs61405042
rs117630647
rs611646
rs6007020
rs2056726
rs34550383
rs142426306
rs11426156
rs2967355
rs1003322
rs4724
rs116863223
rs869785
rs13230646
rs7221585
rs1985369
rs7790856
rs6790988
rs6776756
rs73730598
rs3891167
rs12932179
rs131797
rs111950327
rs11991877
rs137901416
rs4731541
rs28363070
rs1957937
rs35640778
rs429358
rs11584821
rs139669835
rs139795227
rs16978028
rs13062095
rs59409453
rs6587577
rs6536702
rs111527438
rs1291143
rs10112752
rs117512405
rs6590343
rs77732866
rs10905255
rs6584579
rs12412214
rs8102497
rs11557154
rs112394943
rs17445108
rs11769630
rs185174247
rs6881568
rs117407747
rs4498805
rs450962
rs41269079
rs1332941
rs7099229
rs6054257
rs55747751
rs12925933
rs145114957
rs45604339
rs11085072
rs3767952
rs9955360
rs11117354
rs2293579
rs3093888
All − MR Egger
All − Inverse variance weighted
−2
−1
0
1
2
MR effect size for
'telomere length || id:ieu−b−4879' on 'IDP_dMRI_TBSS_L1_Inferior_cerebellar_peduncl
